# Characteristics of squamous cell carcinoma on hidradenitis suppurativa lesions – a case series

**DOI:** 10.1111/ddg.15708

**Published:** 2025-04-03

**Authors:** Nessr Abu Rached, Riina Käpynen, Yannik Haven, Lennart Ocker, Carolin Frost, Eggert Stockfleth, Falk G. Bechara

**Affiliations:** ^1^ International Centre for Hidradenitis suppurativa / Acne inversa (ICH) Department of Dermatology Venereology and Allergology Ruhr‐University Bochum Bochum Germany; ^2^ Skin Cancer Center Department of Dermatology Venereology and Allergology Ruhr‐University Bochum Bochum Germany

**Keywords:** Hidradenitis suppurativa, HS, acne inversa, malignoma, squamous cell carcinoma, HPV

Dear Editors,

Hidradenitis suppurativa (HS) is a chronic inflammatory skin disease that mainly affects the intertriginous regions. Chronic inflammation, viral induction by human papillomavirus (HPV), chronic wound healing deficiency and high tobacco consumption increase the risk of cutaneous squamous cell carcinoma (cSCC).^1^ Overall, cSCC in HS lesion is a rare but very severe complication of HS (Figure 1). Therefore, we collected our cases with cSCC retrospectively to identify characteristics.

**FIGURE 1:**
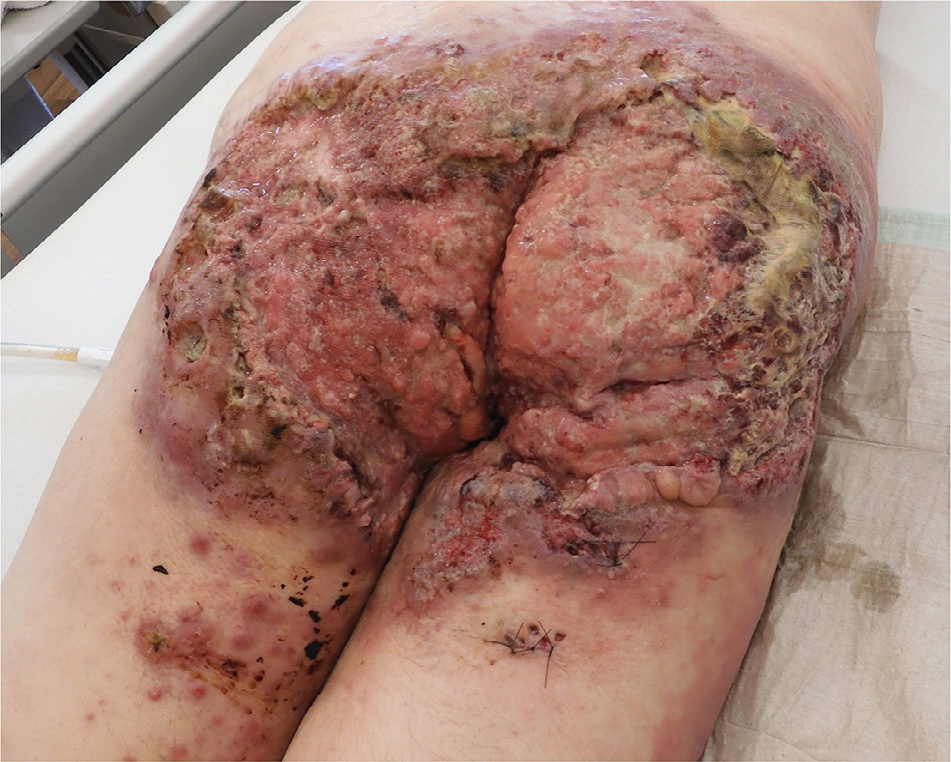
Clinical images of squamous cell carcinoma in hidradenitis suppurativa lesions: There is an ulcerated squamous cell carcinoma infiltrating the entire gluteal and perianal area.

We performed a retrospective analysis on a cohort of seven patients with hidradenitis suppurativa diagnosed with cutaneous squamous cell carcinoma. The cohort was predominantly male (85.7%), with a median age at cSCC diagnosis of 60 years (range: 42–65 years) (Table 1). The median duration of HS at the time of cSCC diagnosis was 28 years (range 14–47), highlighting the prolonged inflammatory burden associated with malignant transformation in these patients. All patients were classified as Hurley stage III. The median BMI was 25.7 kg/m^2^ (range: 21–36.4 kg/m^2^), which is low for a typical HS cohort. Smoking status revealed 42.9% active smokers and 57.1% ex‐smokers, with a median pack‐year history of 30 (range: 0–45). Common locations of HS involvement included the perineal/perianal region (100%), gluteal region (85.7%), and inguinal region (71.4%). HS‐associated lymphoedema was noted in 42.9% of patients, and 57.1% had received biologic pretreatment, specifically secukinumab (14.3%) and adalimumab (42.9%).

**TABLE 1:** Personal characteristics, tumor‐specific, HS‐specific and laboratory characteristics of patients with squamous cell carcinoma on hidradenitis suppurativa lesions (n = 7).

Parameters	Value
***Personal and HS‐specific characteristics***	
Sex, n (%)	
Female	1 (14.3)
Male	6 (85.7)
Age at diagnosis of cSCC, median (range), y	60 (42–65)
Age of HS onset, median (range), y	29.3 (14–51)
Duration of HS at diagnosis of cSCC, median (range), y	28 (14–47)
BMI, median (range), kg/m^2^	25.7 (21–36.4)
Family history of HS, n (%)	
Positive	2 (28.6)
Negative	5 (71.4)
Current smoker status, n (%)	
Active smokers	3 (42.9)^*^
Non‐smokers	0 (0)
Ex‐smoker	4 (57.1)
Tobacco pack years, median (range)	30 (0–45)^*^
Hurley stage, n (%)	
Hurley I	0 (0)
Hurley II	0 (0)
Hurley III	7 (100)
Axillary involvement of HS, n (%)	4 (57.1)
Sub‐ or intermammary involvement of HS, n (%)	1 (14.3)
Genital involvement of HS, n (%)	4 (57.1)
Gluteal involvement of HS, n (%)	6 (85.7)
Inguinal involvement of HS, n (%)	5 (71.4)
Perineal/perianal involvement of HS, n (%)	7 (100)
HS associated lymphoedema, n (%)	3 (42.9)
Biologics pretreatment, n (%)	4 (57.1)
Secukinumab pretreatment, n (%)	1 (14.3)
Adalimumab pretreatment, n (%)	3 (42.9)
Number of affected HS locations, median (range)	4 (3–9)
***Laboratory parameters at baseline (diagnosis of cSCC)***	
CRP in mg/l, median (range)	124.4 (79.9–186.6)
Leukocyte cells/µl, median (range)	12,010 (8,850–41,130)
Thrombocytes/µl, median (range)	398,000 (325,000–565,000)
***Tumor‐specific data***	
Number of cSCC‐specific deaths, n (%)	4 (57.1)
Time to death in months, median (range)	3 (0–34)
Localization of the cSCC, n (%)	
Perianal	2 (28.6)
Gluteal	3 (42.9)
Inner thigh	1 (14.3)
Sacral	1 (14.3)
Tumor differentiation	
Grade 1, n (%)	4 (57.1)
Grade 2, n (%)	2 (28.6)
Grade 3, n (%)	1 (14.3)
Number of patients with metastases, n (%)	3 (42.9)

*Abbr*.: n, absolute number of patients; y, years; BMI, body mass index; HS, hidradenitis suppurativa; cSCC, cutaneous squamous cell carcinoma;

* An HS patient with 60 joint years without tobacco consumption.

Regarding tumor‐specific data, 57.1% of patients died from cSCC, with a median time to death of 3 months (range: 0–34 months). The most common localization of cSCC were the gluteal region (42.9%) and the perianal region (28.6%). The tumor differentiation grades were Grade 1 (57.1%), Grade 2 (28.6%), and Grade 3 (14.3%). Metastases were present in 42.9% of patients. One patient had cutaneous, lymphogenic, pulmonary metastases, one had lymphogenic metastases, and one had bone metastases. The other four patients had no local or distant cSCC metastases.

As already described in the literature, our group also confirmed that cSCC on HS lesions mainly affects men and the lower body region (especially perianal/perineal).^2^ HS patients with high disease severity, as reflected by high inflammatory markers and Hurley III, appear to be particularly affected by cSCC. Notable is the low median BMI of our HS cohort of 25.7 kg/m2 (range 21–36.4). The typical mean BMI of our HS cohort is 31.5 kg/m^2^ (standard deviation ± 6.7).^3^ Tumor‐related weight loss may explain the big difference. BMI was not reported in the previously published case series. Although the reason for the weight loss remains speculative, it is likely to be the main cause.

In general, cSCCs in HS lesions are more aggressive than cSCCs in other sites. This is reflected in a high mortality rate of 57.1% with a median time to death of 3 months (range 0–34). However, it is important to highlight that two of the three patients still alive are newly diagnosed cases of cSCC, so the mortality rate could be even higher. An inflammatory tumor microenvironment may play an important role in explaining the high aggressiveness of cSCC in HS. The inflammatory microenvironment of HS with the cytokines TNF‐α, interleukin‐6, interleukin‐17, and interleukin‐1β could favor tumor development. However, long‐term tobacco consumption should not be underestimated in the development of tumors. Strikingly, all of our patients had smoked for a long time (median of tabacco pack years: 30, range 0–45). Certain compounds in cigarettes and cannabinoids can also damage DNA and promote the development of tumors.^4^, ^5^ Another reason for the poor outcome of HS patients with cSCC may be the difficulty in histological diagnosis of cSCC. The massive inflammation may make it difficult for the pathologist to recognize the diagnosis of cSCC (Figure 2). High levels of CRP are probably related to the severe inflammation in HS. Another possibility is that the high CRP level is to some extent induced by the tumor. Both hypotheses are possible and cannot be verified by the case series.

**FIGURE 2:**
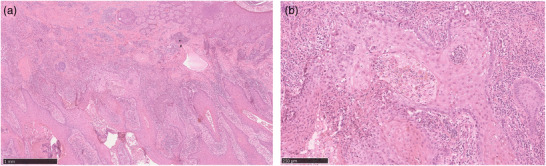
The figure shows a cSCC on HS lesions based on hematoxylin and eosin staining: (a) overview and (b) 100 x magnification. Histologically, keratinocytes and pleomorphic tumor cells infiltrate beyond the basement membrane. A dense lymphocytic infiltrate surrounds the fistula tracts of both HS and cSCC.

Although the importance of HPV as a human epithelial carcinogen is well established in cervical and oropharyngeal cancers, the role of HPV in HS lesions remains unclear. The association between smoking and increased anogenital HPV infection paired with the inflammatory tumor microenvironment in HS may affect and increase the risk for carcinogenesis and the severity of their disease as observed in our cohort.^6^

A limitation of this case series is that the data were collected at a single center. The monocentric nature of the analysis could lead to bias, as Bochum is a referral center for severe cases of HS.

Our cases demonstrate that cSCC is a severe complication of HS with a short time to death. Overall, cSCC in HS lesions occurs in males, smokers, Hurley III patients and in the perineal/gluteal region. Close monitoring of high‐risk HS patients and, if necessary, mapping biopsies should be considered.

## CONFLICT OF INTEREST STATEMENT

N.A. received funding, travel support, and/or personal honoraria for lectures from Novartis Pharma and Johnson & Johnson that were independent of the work submitted. F.G.B. has received honoraria for participation in advisory boards, in clinical trials, and/or as a speaker from AbbVie Inc., AbbVie Deutschland GmbH & Co. KG, Boehringer Ingelheim Pharma GmbH & Co. KG, Novartis Pharma GmbH, UCB Pharma, Incyte Corporation, and Janssen Cilag GmbH, MoonLake. E.S. has received lecture fees from Almirall, Leo, Pierre Favre and Philips. L.O. has received honoraria as a speaker and/or travel support from Novartis Pharma GmbH, Incyte Biosciences Corporation and Janssen. All other authors (Y.H., R.K. and C.F.) declare no conflicts of interest.
